# Efficacy of an attenuated vaccine against avian coccidiosis in combination with feed additives based on organic acids and essential oils on production performance and intestinal lesions in broilers experimentally challenged with necrotic enteritis

**DOI:** 10.1016/j.psj.2022.101848

**Published:** 2022-03-15

**Authors:** Ellen van Eerden, Regiane R. Santos, Francesc Molist, Martina Dardi, Luis Augusto Pantoja-Millas, Joan Molist-Badiola, Massimiliano Baratelli, Marc Pages

**Affiliations:** ⁎ Schothorst Feed Research, Lelystad 8200 AM, the Netherlands; † Laboratorios Hipra S.A., Amer 17170, Spain

**Keywords:** broilers, necrotic enteritis, vaccine, *Eimeria*, feed additives

## Abstract

Several factors predisposing to necrotic enteritis (**NE**) have been identified, including diet and *Eimeria* spp*.* infestations. Coccidiosis vaccines are indicated to decrease the intestinal lesions caused by specific *Eimeria* species that are a known predisposing factor to NE and, consequently, these vaccines could be a holistic approach to the control of NE disease and an alternative solution to coccidiostats. Besides, feed additives have also gained special attention from the poultry industry as an alternative solution to antibiotics to prevent NE as well as other bacterial enteritis.

Then, the combination of vaccination against coccidiosis and the supplementation of the diet with feed additives could be a composite approach to the control of NE problems triggered by *Eimeria* spp. infestation. The objective of this study was to test the efficacy of an attenuated coccidiosis vaccine (EVANT) in combination with different feed additives to prevent the loss of production performance and intestinal lesions in broilers challenged with NE.

Healthy day-old broilers (n = 960) were randomly allocated to 6 groups (8 cages/group). Groups 1–2 were left unvaccinated. Groups 3–6 were vaccinated following the manufacturer's instructions. Chickens were grown using a diet favoring the intestinal proliferation of *Clostridium perfringens*. Moreover, the diets of groups 4–6 were supplemented with medium chain fatty acids (**MCFA**), butyric acid or phytogenic feed additives (**PFA**), respectively. A NE infection model was used to challenge groups 2–6; chickens were orally infected with *Eimeria maxima* (4,500 oocysts) and then *C. perfringens* (10^8^ CFU) at 15 and 20 d, respectively. Birds were monitored and productive parameters recorded until 42 d; intestinal lesions were scored.

Results showed that coccidiosis vaccination, with or without the addition of feed additives, decreased intestinal lesions associated with NE and improved the performance of the birds. Besides, the addition of MCFA to the diet decreased intestinal lesions associated to NE in vaccinated animals compared to all treatment groups. Moreover, the same additive improved the feed conversion rate. Therefore, vaccination with a live attenuated coccidiosis vaccine together with in-feed inclusion of MCFA might be a solution to reduce NE in broilers raised antimicrobial- and coccidiostat-free.

## INTRODUCTION

Necrotic enteritis (**NE**) is an intestinal disease caused by pathogenic toxin-producing *Clostridium perfringens* in the gut (Keyburn et al., 2008). Among its predisposing factors (Moore, 2016) are intestinal damage caused by *Eimeria* spp. and diet including grains that are rich in soluble non-starch polysaccharides (**NSPs**) (Yegani and Korver, 2008). Vaccination against *Eimeria* spp. has been proposed as an alternative solution for controlling coccidiosis, which may also help, at least partially and indirectly, in controlling the burden of NE (Williams et al., 2003). Besides, some feed additives, as short-chain fatty acids (**SCFA**) (Zou et al., 2019), medium-chain fatty acids (**MCFA**) (Yang et al., 2019, Zeitz et al., 2015) and phytogenic feed additives (**PFA**), also appear as potential candidates to minimize the effects of NE (Abdelli et al., 2020, Granstad et al., 2020), instead of using antimicrobial drugs.

Our hypothesis is that prophylaxis by means of an attenuated anticoccidial vaccine (Williams, 2002, Williams et al., 2003) combined with dietary supplementation with organic acids like MCFA (mainly lauric acid), SCFA (coated butyrate), (Dibner and Buttin, 2002) or PFA (phytogenic feed additives –essential oils plus benzoic acid-) (Burt, 2004) supports broilers during and after a challenge with *E. maxima* and *C. perfringens*, thus improving gut health and growth performance (Adhikari et al., 2020).

## MATERIALS AND METHODS

### Ethics Statement

The experiment was approved by the Institutional Animal Welfare Body of Schothorst Feed Research and was conducted according to the restrictions provided by the Animal and Human Welfare Codes of The Netherlands under code AVD246002016776.

### Birds and Housing

A total of 960 one-day-old male Ross 308 broiler chickens housed at the broiler facilities of Schothorst Feed Research (Lelystad, the Netherlands) and reared in two-tier cages at a maximum density of 20 birds per cage. Each cage was equipped with wood shavings as bedding material. The birds had ad libitum access to feed and drinking water. The health status of the flock was monitored daily.

### Experimental Design

Upon arrival at the trial facility, the birds were randomly allocated to one of 6 treatment groups with 8 replicate cages per treatment. The treatment factors were anticoccidial vaccination with EVANT, NE challenge, and dietary supplementation. The six groups and treatments they received are presented in Table 1.

**Table 1:** Description of treatments and diet codes.

Treatment	Description	Vaccine	NE challenge	Feed additive	Feed additive dose
1	Negative control (NC)	NO	NO	NO	-
2	Positive control (PC)	NO	YES	NO	-
3	Vaccine (VO)	YES	YES	NO	-
4	Vaccine + MCFA (VM)	YES	YES	Lauric acid 47%	15 g/ Kg
5	Vaccine + SCFA (VS)	YES	YES	Butyric acid	1.5 g/kg
6	Vaccine + PFA (VP)	YES	YES	Essential oils (thymol, eugenol, piperine) and benzoic acid	0.3 g/kg

### Diet

The diet was formulated according to a 3-phase feeding program to meet or exceed the birds’ nutritional requirements (CVB, 2018). The feeding phases were a starter phase from d 0 to 14, a grower phase from d 14 to 28, and a finisher phase from d 28 to 42. The feed compositions are presented in Table 2. The grower diet was specifically formulated to provide suitable conditions to promote the development of NE, meaning that feedstuffs rich in soluble NSPs were used, without using enzymes like xylanase and glucanase. One batch remained nonsupplemented and was supplied to the **NC, PC, and VO** treatments. The other three sub-batches were supplemented with MCFA (Grolux Synergy) at 15 g/kg, SCFA (Adimix Precision) at 1.5 g/kg, or essential oils blended with benzoic acid (CRINA Poultry Plus) at 0.3 g/kg, and were supplied to the VM, VS, and VP treatments, respectively. The active substances in Grolux Synergy were mainly C12:0 (47%) and C14:0 (18%) fatty acids (and other fatty acids adding up to 100%), Adimix Precision contained 30% of coated sodium butyrate, and CRINA Poultry Plus contained 80% benzoic acid and a blend of approximately 1.5% of thymol, eugenol, and piperine. Due to the energy contribution of the fatty acids in Grolux Synergy and in order to maintain isocaloric diets across treatments, Grolux Synergy was balanced with poultry fat in the diet. No anticoccidial drugs were used in the diets.

**Table 2:** Composition of the experimental diets per feeding phase.

Ingredients (%)	Starter d 0–14(0-14 d)	Grower d 14–28 (14-28 d)	Finisher d 28–42(28-42 d)
Wheat	41.679	37.022	49.171
Barley	—	25.000	—
Maize	20.000	—	20.000
Soybean meal	27.120	24.012	23.407
Rapeseed meal	4.000	—	—
Toasted full-fat soybeans	—	5.025	—
Poultry fat	3.119	3.833	4.347
Soybean oil	—	1.917	—
Limestone	1.114	0.951	0.976
Monocalcium phosphate	0.549	0.278	0.121
Salt	0.370	0.279	0.212
Lysine-HCI (L 79%)	0.269	0.247	0.253
Methionine (DL 99%)	0.264	0.263	0.212
Threonine (L 98%)	0.098	0.097	0.086
Valine (L 99%)	0.068	0.053	0.050
Glucanase/xylanase premix^1^	0.250	—	0.250
Sodium bicarbonate	—	0.100	0.160
Vitamin & mineral premix	0.600	0.500	0.400
Phytase	0.500	0.423	0.354
			
Calculated nutrients (g/kg)			
AMEn, kcal/kg	2,850	2,900	3,025
DM	878.8	882.4	878.5
Ash	51.4	46.0	39.9
Crude protein	214.8	211.0	191.1
Crude fat (acid hydrolysis)	57.2	89.5	68.5
Crude fiber	27.2	29.5	22.7
Ca	6.46	5.19	4.81
P	5.01	4.21	3.6
ret.P	3.8	3.2	2.7
K	9.1	9.1	8.0
Na	1.6	1.5	1.4
Cl	3.4	2.8	2.3
AFD LYS	11.5	11.0	10.0
AFD MET	5.50	5.27	4.65
AFD M+C	8.43	8.06	7.30
AFD THR	7.48	7.15	6.5
AFD TRP	2.32	2.30	2.06

1 Rovabio Exce.

### Coccidiosis Vaccine

EVANT (Laboratorios HIPRA S.A., Spain) is a vaccine for preventing avian coccidiosis. It is formulated with live attenuated *Eimeria* spp. oocysts and the adjuvanted solvent HIPRAMUNE T. The vaccine composition is as follows: oocysts of *Eimeria acervulina* (Strain 003, 332–450 oocysts per dose), *E. maxima* (Strain 013, 196–265 oocysts per dose), *E. mitis* (Strain 006, 293–397 oocysts per dose), *E. praecox* (Strain 007, 293–397 oocysts per dose), and *E. tenella* (Strain 004, 276–374 oocysts per dose). HIPRAMUNE T contains an immunostimulant adjuvant and other excipients designed to stimulate preening behavior during spray vaccination. EVANT was administered to the day-old birds in the VO, VM, VS, and VP treatments by means of coarse spray following the manufacturer's instructions. The NC and PC treatments were sham-vaccinated by administering a coarse spray of phosphate buffered saline (**PBS**).

### *Eimeria maxima* and *Clostridium perfringens*

Sporulated *E. maxima* oocysts (Weybridge strain) and pathogenic α-toxin and NetB toxin producing *C. perfringens* were provided by Royal GD (Deventer, the Netherlands).

The *C. perfringens* inoculum consisted of an overnight culture that was grown in liver broth provided by BioTrading Benelux B.V., and the bacterial load was determined by plating serial dilutions of the inoculum on Sheep Blood Agar. On d 15, birds in the **NC** group were orally sham-inoculated with 1 mL of PBS, whereas all other birds were orally inoculated with 1 mL of PBS containing 4,500 sporulated oocysts of *E. maxima*. PBS was freshly prepared by dissolving 10.26 g Na2HPO4.2H2O, 2.36 g KH2PO4, and 4.5 g NaCl in 1 L of demineralized water. After adjusting the pH to 7.2, the PBS was sterilized by autoclaving. On d 20, birds in the **NC** group were orally sham-inoculated with 1 mL of sterile liver broth, whereas all other birds were orally inoculated with 1 mL of liver broth containing 2.5 × 10^8^ colony forming units (**cfu**) of *Clostridium perfringens*.

### Measurements

#### Lesion Scoring

Intestinal lesions in birds were scored on d 21 and d 22. For this purpose, four randomly selected birds per cage were euthanized in a CO_2_ chamber. *E. maxima* associated lesions were scored according to Johnson and Reid (1970). Briefly, birds without lesions were scored ‘0’ and birds with *E. maxima* lesions were categorically scored between ‘1’ (mild lesions) and ‘4’ (severe lesions). *C. perfringens* associated lesions were also categorically scored as ‘0’ (no lesions), ‘1’ (1–5 small white lesions, less than 1 mm in diameter), ‘2’ (> 5 small white lesions, less than 1 mm in diameter), ‘3’ (> 5 larger lesions, 1–2 mm in diameter; or erosive zones), or ‘4’ (dead birds with positive NE diagnoses postmortem), as described by Lensing et al. (2010).

#### Production Performance

Birds and feed were weighed on a per cage basis on d 0, 14, 28, and 42. Mortality was daily recorded throughout the experiment. Body weight gain (**BWG**), feed intake (**FI**), and feed conversion ratio (**FCR**) were calculated for each feeding phase and for the overall study period.

### Statistical Analysis

Performance raw data and OPG counts were analyzed by analysis of variance, using the model: Y_ij_ = µ + Block_i_ + Treatment_j_ + e_ij_ (Y_ij_ = dependent variable; µ = overall mean; Block_i_ = block effect [i = 1…8]; Treatment_j_ = effect of treatment group [j = 1…6]; e_ij_ = residual error). Fisher's post-hoc Least Significant Difference (**LSD**) was used to identify differences between treatment groups. Values with *P* ≤ 0.05 were considered statistically significant. Statistical analysis was based on two-sided tests.

## RESULTS

### Intestinal Lesions

*E. maxima* lesions were present on d 21 and d 22 in the challenged treatments, but they were mild, with significant treatment differences only on d 21 (Table 3). The VM treatment was the only vaccinated treatment with a lower coccidiosis score (*P* < 0.05) than the PC treatment on d 21. There was no further decrease in coccidiosis lesions in the VS and VP treatments compared to the VO treatment. In contrast, rather severe *Clostridium* lesions were present in the PC treatment on both days, with all vaccinated treatments having lower *Clostridium* scores than the PC treatment (*P* < 0.05). On d 22, the *Clostridium* lesions in the VM treatment were lower compared to the VO treatment (*P* < 0.05), but the VS and VP treatments were not further decreased compared to the VO treatment.

**Table 3:** Mean intestinal lesion scores on d 21 and d 22, as affected by treatment.

Treatment	d 21				d 22		
	Coccidiosis		Clostridium		Coccidiosis	Clostridium	
NC	0	a	0	a	0	0	a
PC	0.63	c	2.16	d	0.13	2.13	c
VO	0.53	bc	0.81	bc	0.16	0.91	b
VM	0.28	ab	0.38	ab	0.09	0.41	a
VS	0.44	bc	1.03	c	0.03	0.97	b
VP	0.66	c	1.03	c	0.03	0.91	b
*P*-value	< 0.001		< 0.001		0.12	< 0.001	
LSD	0.301		0.489		0.127	0.485	

a-d Values without a common superscript in a column differ significantly (*P* < 0.05). Abbreviations: N, negative control (nonvaccinated, noninfected, nonsupplemented); PC, positive control (nonvaccinated, infected, nonsupplemented); VO, vaccine only (vaccinated, infected, nonsupplemented); VM, Vaccine + MCFA (vaccinated, infected, supplemented with medium-chain fatty acids); VS, Vaccine + SCFA (vaccinated, infected, supplemented with short-chain fatty acids); VP, Vaccine + PFA (vaccinated, infected, supplemented with essential oils plus benzoic acid).

### Growth Performance

Body weight (**BW**) and FI were periodically recorded during the experimental period (Table 4) to investigate the efficacy of the vaccination and the feed additives on production performance.

**Table 4:** Effect of treatments on body weight (BW; g), body weight gain (BWG; g), feed intake (FI; g), and feed conversion ratio (FCR; g/g).

	Treatments													
Productive parameters	NC		PC		VO		VM		VS		VP		*P*-value	LSD
**d 0–14**														
BW d 14	534	c	535	c	489	ab	477	a	494	b	484	ab	< 0.001	14.7
BWG (g)	491	c	492	c	446	ab	434	a	455	b	441	a	< 0.001	14.1
FI (g)	550	c	554	c	520	b	495	a	528	b	513	b	< 0.001	16.3
FCR (g/g)	1.121	a	1.126	a	1.167	c	1.141	b	1.160	c	1.162	c	< 0.001	0.0130
**d 14–28**														
BW d 28	1,743	c	1,630	a	1,691	abc	1,696	bc	1,678	ab	1,650	ab	0.02	62.3
BWG (g)	1,210	b	1,095	a	1,203	b	1,219	b	1,184	b	1,167	b	0.003	59.6
FI (g)	1,721		1,727		1,776		1,737		1,762		1,720		0.64	79.7
FCR (g/g)	1.423	a	1.581	c	1.476	b	1.424	a	1.488	b	1.474	b	< 0.001	0.0267
**d 28–42**														
BW d 42	3,440		3,382		3,397		3,376		3,400		3,421		0.90	119.8
BWG (g)	1,718		1,752		1,750		1,680		1,721		1,771		0.27	80.2
FI (g)	2,817	b	2,899	b	2,843	b	2,688	a	2,817	b	2,792	ab	0.03	114.7
FCR (g/g)	1.640	bc	1.654	c	1.625	abc	1.602	ab	1.637	bc	1.578	a	0.04	0.0476
**d 0–42**														
BWG (g)	3,397		3,340		3,354		3,333		3,357		3,378		0.90	119.8
FI (g)	5,092		5,181		5,072		4,919		5,100		5,040		0.16	187.7
FCR (g/g)	1.499	abc	1.551	d	1.512	bc	1.476	a	1.519	c	1.492	ab	< 0.001	0.0265

a-d Values without a common superscript in a column differ significantly (*P* < 0.05). Abbreviations: NC, negative control (nonvaccinated, noninfected, nonsupplemented); PC, positive control (nonvaccinated, infected, nonsupplemented); VO, vaccine only (vaccinated, infected, nonsupplemented); VM, Vaccine + MCFA (vaccinated, infected, supplemented with medium-chain fatty acids); VS, Vaccine + SCFA (vaccinated, infected, supplemented with short-chain fatty acids); VP, Vaccine + PFA (vaccinated, infected, supplemented with essential oils plus benzoic acid).

The NE challenge in the period between d 14 and d 28 resulted in a lower BWG (*P* < 0.05) and higher FCR (*P* < 0.05) in the PC treatment compared to all other treatments, while FCR in the VO, VS, and VP treatments was higher (*P* < 0.05) than in the NC and VM treatments. Considering the overall experimental period, that is, d 0 to 42, the NC and all vaccinated treatments had a lower FCR (*P* < 0.05) than the PC treatment, but there were no significant treatment differences in BWG and FI. The vaccinated treatments did not differ from the NC treatment on overall FCR (*P* > 0.05), but among the vaccinated treatments, the VM treatment was the only one with an improved FCR (*P* < 0.05) compared to the nonsupplemented VO treatment. There were no significant differences in mortality between treatments.

## DISCUSSION

This experiment was conducted following the hypothesis that the combination of an anticoccidial vaccine with feed additives based on MCFA, SFCA, or PFA would support broilers on gut health and production performance during and after an NE challenge. The NE challenge was induced by a predisposing diet, a primary challenge with a non-attenuated *E. maxima* strain on d 15 and a secondary challenge with an α-toxin and NetB toxin producing *C. perfringens* strain on d 20.

The NE challenge in the present study produced intestinal lesions mainly associated with *C. perfingens* and a decrease in production performance. These results were similar to what was previously reported for subclinical NE in broilers (Skinner et al., 2010). However, administration of the anticoccidial vaccine, with or without feed additives, significantly reduced the lesions caused by *C. perfringens*, and thus greatly contributed to control of intestinal damage due to NE. Moreover, the vaccine reduced the loss of BWG and FCR by 94 and 66%, respectively, relative to the NC treatment. These results indicate that protection against coccidiosis, one of the predisposing factors of NE, contributes to reducing *C. perfringens* lesions and the collateral effects on FCR.

The active components in the feed additives tested in this study were reported to have antimicrobial activity (Jozefiak et al., 2010; Timbermont et al., 2010), but it was unknown whether their addition to the diet could provide any further benefit to controlling NE in addition to vaccination. Lauric acid is a C12 fatty acid and was the major component (nearly 50%) in the MCFA feed additive in our study. Lauric acid is reported to improve BWG and FCR (Zeitz et al., 2015), but it has also in vitro antimicrobial activity against *C. perfringens* (Skrivanova et al., 2005). Furthermore, it has been shown to reduce the incidence of intestinal lesions in broilers challenged with NE (Timbermont et al., 2010), although the effects on intestinal lesion levels are sometimes contrasting.

In the present study, it was shown that 1.5% of dietary MCFA throughout the study period, combined with the anticoccidial vaccine, was effective in minimizing the negative impact of NE. Not only did it decrease the severity of the intestinal lesions, but also improved the birds’ performance, with the most pronounced effect on FCR. Moreover, it also improved FCR before the start of the challenge period, when a mild vaccination response in the other vaccinated treatments was reflected in a higher FCR. As the MCFA was not tested alone, that is, without vaccination, it is not possible to establish whether the observed effects were additive or synergistic to the effect of the vaccine.

The addition of SCFA and PFA to the diet of vaccinated birds did not provide any further reduction of intestinal lesions or improvement in FCR compared to the nonsupplemented vaccinated birds.

## CONCLUSIONS

It can be concluded from this study that a live attenuated vaccine against avian coccidiosis can reduce the severity of intestinal lesions and the loss in BWG and feed efficiency caused by an experimental NE model. Moreover, the supplementation of a feed additive based on C12 and C14 fatty acids in the feed of vaccinated birds improved the abovementioned benefit on FCR compared to nonsupplemented vaccinated birds.
